# How should radiation exposure be handled in fluoroscopy‐guided endoscopic procedures in the field of gastroenterology?

**DOI:** 10.1111/den.14208

**Published:** 2022-01-12

**Authors:** Mamoru Takenaka, Makoto Hosono, Shiro Hayashi, Tsutomu Nishida, Masatoshi Kudo

**Affiliations:** ^1^ Departments of Gastroenterology and Hepatology Kindai Osaka Japan; ^2^ Department of Radiology Kindai University Faculty of Medicine Osaka Japan; ^3^ Department of Gastroenterology and Internal Medicine Hayashi Clinic Osaka Japan; ^4^ Department of Gastroenterology Toyonaka Municipal Hospital Osaka Japan

**Keywords:** fluoroscopy‐guided endoscopic procedures, radiation exposure, radiation exposure protection

## Abstract

Fluoroscopy‐guided endoscopic procedures (FGEPs) are rapidly gaining popularity in the field of gastroenterology. Radiation is a well‐known health hazard. Gastroenterologists who perform FGEPs are required to protect themselves, patients, as well as nurses and radiologists engaged in examinations from radiation exposure. To achieve this, all gastroenterologists must first understand and adhere to the International Commission on Radiological Protection Publication. In particular, it is necessary to understand the three principles of radiation protection (Justification, Optimization, and Dose Limits), the As Low As Reasonably Achievable principle, and the Diagnostic Reference Levels (DRLs) according to them. This review will mainly explain the three principles of radiation exposure protection, DRLs, and occupational radiological protection in interventional procedures while introducing related findings. Gastroenterologists must gain knowledge of radiation exposure protection and keep it updated.

## Introduction

In the field of gastroenterology, various fluoroscopy‐guided endoscopic procedures (FGEPs) such as balloon‐assisted enteroscopy, gastrointestinal metallic stent placement, endoscopic retrograde cholangiopancreatography (ERCP), and endoscopic ultrasound (EUS)‐guided drainage are rapidly gaining popularity, and it is well known that radiation is involved in health hazards.^1^, ^2^, ^3^, ^4^, ^5^, ^6^, ^7^, ^8^, ^9^, ^10^, ^11^ In the past, various FGEPs have been evaluated based on the rates of procedural success, clinical success, and complications, but radiation exposure has not been adequately evaluated, suggesting that gastroenterologists have lacked sufficient awareness regarding radiation exposure.

**Figure 1:**
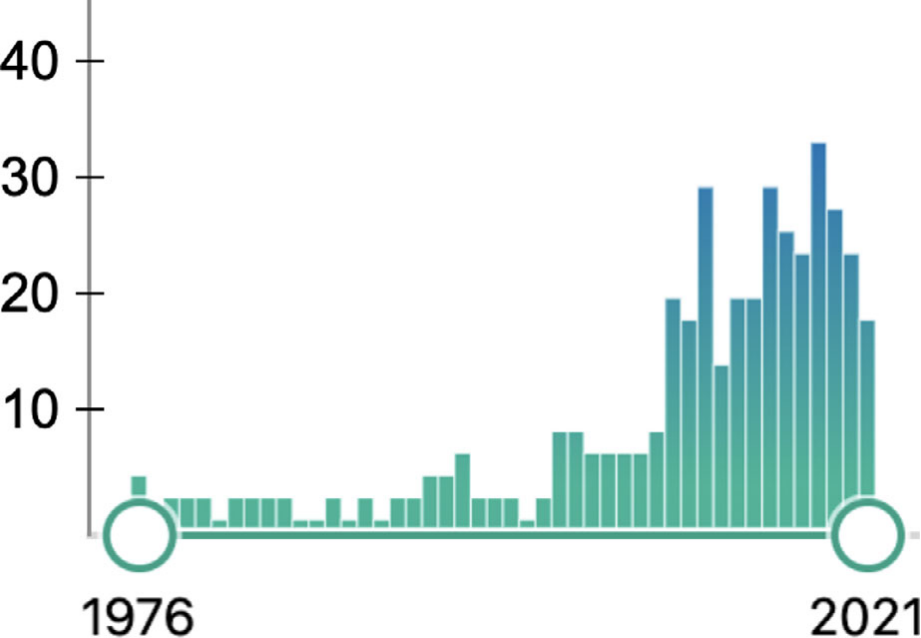
The number of papers obtained in PubMed using the key words “radiation exposure” and “endoscopic retrograde cholangiopancreatography”. The number of papers has increased rapidly in the last 10 years.

Recently, however, radiation dose has been used in comparative study of FGEPs.^12^, ^13^ With the increasing number of reports on FGEPs and radiation exposure, gastroenterologists' awareness status on radiation exposure is gradually changing (Fig. 1).

Radiation exposure includes patient exposure and occupational exposure, and gastroenterologists are obliged to make maximum efforts to protect not only patients and themselves but also medical staff. This review will summarize what is known about radiation exposure and what protection measures should be taken so that gastroenterologists can appropriately handle radiation exposure in FGEPs.

## ICRP Publication

For gastroenterologists to gain knowledge about radiation exposure, it is recommended that the existence of the International Commission on Radiological Protection (ICRP) publication is first recognized. The ICRP is an independent, international, nongovernmental organization that aims to protect people, animals, and the environment from the harmful effects of radiation.

The key function of the ICRP has been to issue recommendations in the form of reports and publications, with their contents being constantly updated and periodically published as “Publication.” Some of these issues contain information on medical radiation exposure protection, and many can be downloaded for free from the ICRP website (Table 1).^14^, ^15^, ^16^, ^17^, ^18^, ^19^, ^20^

**Table 1:** ICRP publications to be referenced by gastroenterologists

Publication number	Year	Title
139	2018	Occupational radiological protection in interventional procedures
135	2017	Diagnostic reference levels in medical imaging
130	2015	Occupational intakes of radionuclides
117	2010	Radiological protection in fluoroscopically guided procedures outside the imaging department
105	2007	Radiological protection in medicine
103	2007	The 2007 recommendations of the international commission on radiological protection
85	2000	Avoidance of radiation injuries from medical interventional procedures
1	1959	Recommendations of the International Commission on Radiological Protection (1st)

ICRP, International Commission on Radiological Protection.

This review will explain the three principles of radiation exposure protection described in Publication 103,^15^ Diagnostic Reference Levels (DRLs) described in Publication 135,^20^ and occupational radiological protection in interventional procedures described in Publication 139,^16^ while introducing related findings.

## Justification, Optimization, and Dose Limits

The three basic principles of radiation protection by the ICRP are Justification, Optimization, and Dose Limits. To properly handle radiation exposure in FGEPs, these three principles should be kept in mind when considering indications. Justification is the general principle that radiation should be used only if the benefits to the patient outweigh the risks of radiation. Optimization is to keep the individual radiation dose and the number of people involved in fluoroscopy as low as reasonably achievable, taking into account economic and social factors. For FGEPs, the radiation dose must be sufficient to ensure that the procedure is safely performed for the patient through sufficient imaging quality. The ICRP recommends that medical radiation exposure should be as low as reasonably achievable.^15^ This principle is called As Low As Reasonably Achievable (ALARA). Dose limit sets the usage limit for each purpose of radiation use, and as a result, it leads to the reduction of radiation dose. However, dose limit does not apply to medical exposure. This is because applying dose limits to medical exposure may hamper delivery of necessary tests and treatments, thus impairing patient benefits.

Diagnostic Reference Levels is a concept that adheres to these three principles, and radiation must be used while referring to DRLs.

## DRLs

As an adjunct to the concept of ALARA, the ICRP introduced DRLs in 1996 as a standard for medical radiation use.^21^

**Figure 2:**
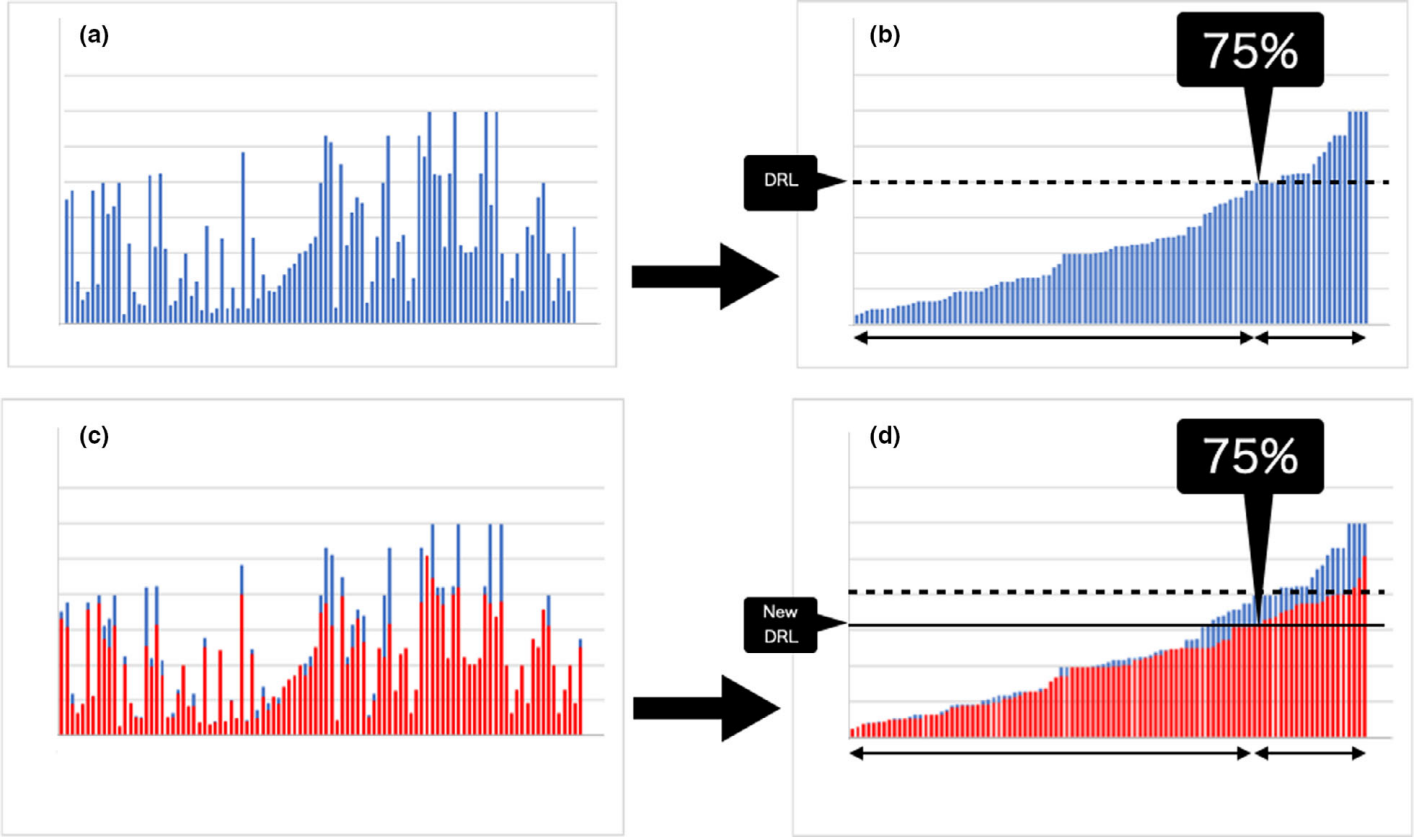
A schema explaining the concept of diagnostic reference levels (DRLs). (a) The setting of DRLs needs the measurement and collection of radiation doses used in the target radiation procedure at as many facilities as possible. Even for the same radiation procedure, the used radiation doses differ from facility to facility; thus, the measured radiation dose values will vary, as shown. (b) To set DRL, these values are sorted in the order of size as shown in (b), and 75% of the total values are set as DRL. (c) If efforts are made to reduce the radiation dose at each facility to reflect this standard value, it will lead to a reduction in the overall radiation dose as shown in (c). (d) This reduction can make the new 75% value the new DRL, as shown in (d), leading to further reductions in overall radiation dose. This process is the concept of DRLs.

The DRL is set at the 75th percentile of radiation dose distribution collected from a particular facility. If efforts are made to adhere to the DRL at each facility reflecting this standard value, it will lead to a reduction in the overall radiation dose. This reduction can result in new values at the 75th percentile of a distribution as new DRLs, leading to further reductions in the overall radiation dose. This process is the concept of DRLs (Fig. 2). ICRP 105 emphasizes the importance of DRLs, and ICRP 135 recommends that all individuals involved in procedures subjecting a patient to a medical exposure should be familiar with the DRL process as a tool for optimizing protection.^14^, ^20^

Diagnostic Reference Level is now recognized as the global standard for fluoroscopy‐guided procedures. Since DRL values vary from country to country even for the same procedure, the DRL guidelines have been updated in each country by their individual radiological societies.

In the United States, the American College of Radiology reported DRLs but did not include DRLs for FGEPs. Regarding ERCP, the American Gastroenterological Endoscopy Society recommends that fluoroscopy time (FT) and radiation dose be used as quality indicators for ERCP, but does not mention DRLs.^22^ In Europe, although the UK has national DRLs, FGEPs are not included. The European Society of Gastrointestinal Endoscopy (ESGE) guidelines on radiation protection in digestive endoscopy recommend the establishment of DRLs for ERCP; however, they referred to a small sample size.^23^

In Japan, the first DRLs were established in 2015 and included six categories: Computed Tomography, General Radiography, Mammography, Dental Radiography, Nuclear Medicine, and IVR, and the category covering FGEPs in the field of gastroenterology was not included.^24^ Factors that influence radiation dose used in these DRLs are air kerma (K_a,r_: mGy) and kerma‐area product (P_KA_: Gy·cm^2^). ICRP Publication 135 recommends that DRLs should be revised at least every 3–5 years, and it was last revised in 2020, with the addition of Diagnostic Fluoroscopy to the category. As shown in Table 2, DRLs for Diagnostic Fluoroscopy list DRLs of FGEPs in the field of gastroenterology such as barium swallow, upper gastrointestinal fluoroscopy, ileus tube insertion, barium enema, and ERCP (diagnostic/treatment).^25^ Among them, the DRL of upper gastrointestinal fluoroscopy with contrast (detailed examination) is the highest, followed by ERCP (treatment) and ileus tube insertion. The DRL of ERCP (diagnostic) is approximately half that of ERCP (treatment).

**Table 2:** DRLs for diagnostic fluoroscopy in DRL Japan 2020

	K_a,r_ (mGy)	P_KA_ (Gy·cm^2^)	FT (min)	No. of images per examination
Barium swallow	30	17	5	5
Upper gastrointestinal fluoroscopy with contrast	110	45	6	27
Upper gastrointestinal fluoroscopy with contrast (detailed examination)	230	61	13	45
Upper gastrointestinal fluoroscopy with contrast (medical checkup)	89	29	6	21
Ileus tube insertion	150	47	28	6
Barium enema	130	46	11	27
ERCP (diagnostic)	93	26	14	12
ERCP (treatment)	170	36	17	13
Bronchoscopy	38	8	8	1
Total parenteral nutrition catheterization (CV catheter‐port insertion)	8	3	3	2
Lumbar nerve root block	49	9	3	2
Lumbar myelography	69	26	4	11

CV, central catheter; DRLs, diagnostic reference levels; ERCP, endoscopic retrograde cholangiopancreatography; FT, fluoroscopic time; K_a,r_, air kerma; P_KA_, kerma‐area product.

Gastroenterologists should handle radiation with reference to these DRLs so that the reference value is reduced at the time of the next revision.

## Patient Radiation Exposure in FGEPs

Patient radiation exposure is the direct exposure of the X‐ray beam. The radiation dose used in FGEPs is directly proportional to patient radiation exposure. Among FGEPs, there are few reports of radiation exposure in balloon‐assisted enteroscopy and gastrointestinal metallic stent placement. Reports from a single facility indicate that the respective median P_KA_, K_a,r_, and FT were as follows: balloon‐assisted enteroscopy, 43 mGy; 22.4 Gy·cm^2^; and 18.2 min and gastrointestinal metallic stent placement, 62 mGy; 12.4 Gy·cm^2^; and 10.4 min.^26^ For further data collection, a prospective multicenter observational study is being conducted. A multicenter prospective radiation dose measurement study in FGEPs (REX‐GI study) is currently ongoing, and the analysis results are awaited.^27^

### Patient radiation exposure in ERCP

Within FGEPs, radiation exposure during ERCP has been evaluated and reported since the early days of its development. In early 1980, Oi *et al*.,^28^ who contributed to the development of ERCP, reported on a remote‐controlled contrast medium injector and stated that “radiation exposure in ERCP should be minimized as much as possible.” However, it is difficult to assess the radiation exposure in ERCP, because ERCP has diverse uses that include diagnostic and therapeutic procedures. It is desirable to categorize the values for diagnostic and therapeutic ERCP as these radiation exposure values differ widely among the two groups.^29^ It has been reported that therapeutic ERCP is associated with significantly higher values of radiation exposure than diagnostic ERCP.^30^ The ESGE guidelines report that the mean value of radiation dose with therapeutic ERCP is three times higher than that with diagnostic ERCP.^23^ Depending on the target disease of ERCP, proximal malignant biliary obstruction (MBO) was reported to require a significantly longer procedure time (PT) and FT and resulted in a greater radiation dose than distal MBO and common bile stones.^29^ However, there is still a lack of disease‐specific and procedure‐specific radiation exposure data in ERCP. Analysis of a prospective multicenter observational study is needed to improve the radiation standards for use of ERCP described in the DRLs Japan mentioned above.^25^, ^27^

### Patient radiation exposure in EUS‐guided drainage

Endoscopic ultrasound‐guided drainage (EUS‐D) has recently gained popularity as a drainage treatment for pancreatobiliary diseases.^3^, ^4^, ^5^, ^6^, ^7^, ^31^, ^32^ Although this procedure uses fluoroscopy, it also uses ultrasound images, and thus, the radiation dose is expected to be lower. However, a study to assess radiation exposure in EUS‐D compared with trance‐papillary biliary drainage by ERCP reported that EUS‐D has a significantly shorter PT but a significantly longer FT and higher radiation exposure.^13^ The subjects of EUS‐D are diverse, including the biliary tract, gallbladder, pancreatic duct, and infectious pancreatic cyst, and EUS‐D has a wide range of procedural difficulties. There is a large gap in difficulty between EUS‐CD for large pancreatic cysts and EUS‐PD for small pancreatic ducts, and it is expected that there will be differences in radiation exposure. Although it was reported that no difference in radiation exposure was observed between each EUS‐D procedure,^13^ the analysis of a prospective multicenter observational study is warranted.^27^

## Occupational Radiation Exposure in FGEPs

In FGEP examinations, the endoscopists, assistant endoscopists, nurses, radiologists, and anesthesiologists in some facilities are all exposed to radiation.

The major source of occupational exposure in FGEPs is the scattered radiation from the patient, rather than the primary X‐ray beam itself. Scattered radiation exposes the eye lens, thyroid gland, and fingers of the medical staff.

This lens exposure dose limit was revised to an average of 20 mSv/year for 5 years and did not exceed 50 mSv in any 1 year in ICRP 118 in 2011^18^ and was so revised in Japan in 2021. It is about one‐seventh of the previous value, and the lens exposure dose of an endoscopist who does not take exposure protection measures can easily exceed the limit. A systematic, methodologic review reported that cumulative doses of up to 100–200 mSv from low‐dose radiation sources do not increase the risk of cancer, but effects occur in the lens at as little as 20 mSv.^33^

Occupational exposure dose is greatly influenced by the location of the medical staff and the type of X‐ray units.

### Influence of the location of the medical staff

In general, medical staff positioned closest to the X‐ray unit receive the highest radiation exposure. If the endoscopist is closest to the X‐ray unit, the occupational exposure of the endoscopist will be the highest among the medical staff. The annual average lens radiation dose of 34 physicians engaged in angiography and interventional radiology procedures at 18 medical facilities in Japan was reported to be approximately eight times higher than that of other healthcare professionals.^34^ If the patient's body movements are large due to instability of sedation, the nurse will be positioned closest to the patient, leading to the highest radiation exposure. The average radiation doses of endoscopists, first assistants, second assistants, and nurses in ERCP were reportedly 340.9, 27.5, 45.3, and 33.1 μSv, respectively, when protective curtains were not used, and that the exposure dose of endoscopists standing closest to the fluoroscopy equipment was approximately 10 times higher than that of other occupations.^35^

### Influence of the type of X‐ray units

There are two main types of X‐ray units: an under‐couch tube type and an over‐couch tube type and the differences between them are summarized in Table 3. The difference is in the location of the X‐ray emitting part, which is either above or below the examination table. Currently, an under‐couch tube type always has a C‐arm function, but some models of an over‐couch tube type do not have a C‐arm function. Models without the C‐arm function are cheaper than models with the C‐arm function. On the other hand, the workspace of an under‐couch tube type is smaller than that of an over‐couch tube type. Since both types have their own pros and cons, either is used depending on the facility, but it is necessary to pay close attention to the difference in terms of radiation exposure.

**Table 3:** Comparison of X‐ray unit (under‐couch tube type and over‐couch tube type)

	Under‐couch tube type	Over‐couch tube type
X‐ray emitting part	Under the fluoroscopy table	Over the fluoroscopy table
Location of X‐ray detectors	Over the fluoroscopy table	Fixed in the fluoroscopy table
C‐arm function	Existence	Existence (depends on model)
Distance between X‐ray emitting part and examination table	Shorter	Longer
Workspace	Smaller	Larger
Patient radiation exposure	Slightly more	Slightly less
Site where scattered radiation hits medical personnel	Lower body	Upper body (lens, neck, head)
Occupational radiation exposure	Less	More
Pros	Flexible X‐ray directions The X‐ray emitting part can be held close to the patient, resulting in better image quality The short distance between the X‐ray emitting part and the X‐ray detector reduces the X‐ray output Less occupational radiation exposure	Easy to touch patients and perform procedures Less likely to cause accidents when the device is moved Image quality is stable Cheaper than C‐arm devices Versatile applications
Cons	The proximity between the patient and the X‐ray detector can interfere with the manipulation The farther the distance between the patient and the X‐ray detector, the lower the image quality The fluoroscopy table is narrow C‐arms are expensive and not very versatile	Limited X‐ray directions High exposure to the head and neck of the physician or technician performing the procedure nearby

**Figure 3:**
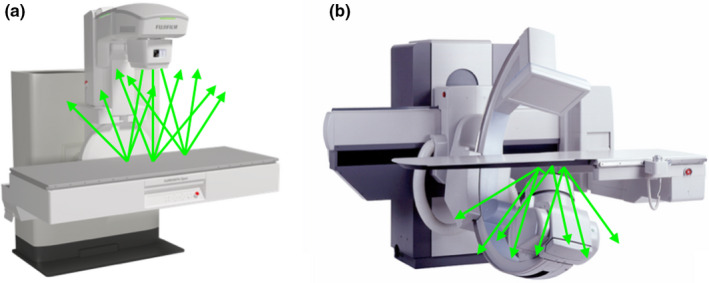
Types of X‐ray units and their respective scattered radiation. (a) Over‐couch tube X‐ray unit. Scattered radiation (arrows) is emitted on the upper side of the endoscopist. (b) Under‐couch tube X‐ray unit. Scattered radiation (arrows) is emitted on the lower body side of the endoscopist.

The scattered radiation is irradiated to the lower body of the medical staff with the under‐couch type and to the upper body with the over‐couch type (Fig. 3). Therefore, an under‐couch tube type can be used to reduce occupational radiation exposure to the lens, thyroid gland, and fingers. Although typical radiation exposure doses are 94 and 75 μGy for the eye and neck, respectively, with an over‐couch type X‐ray unit, the eye and neck doses increase to as high as 550 and 450 μGy, with maximal doses up to 2.8 and 2.4 mGy per procedure, respectively.^36^, ^37^ Additionally, an under‐couch tube X‐ray unit with C‐arm function achieves markedly lower occupational and patient radiation doses than an over‐couch tube X‐ray unit without C‐arm function.^36^

Gastroenterologists who perform FGEPs using an over‐couch tube type must make efforts to adequately protect against radiation exposure, not only for themselves but also for their staff.

## Occupational Radiological Protection in FGEPs

The three basic principles of radiation protection by the ICRP are Justification, Optimization, and Dose Limits as described above, and another three principles of occupational radiological protection when actually using radiation are distance, time, and shielding.^38^

### Distance

With respect to distance, the longer the distance from the X‐ray unit, the lower the radiation exposure. A study measuring radiation doses at distances of 30, 60, 90, 120, 150, and 180 cm from the centerline of an X‐ray beam revealed that the distance was directly proportional to the radiation dose.^39^ This is supported by the lower radiation dose of the assistant endoscopist than that of the endoscopist, as the assistant endoscopist stands farther from the X‐ray unit than the endoscopist.^40^ It is also reported that approximately 85% of the 35 interventionalists who performed IVR for many years and who have developed head and neck cancer have cancer on the left side of the head.^41^

It is necessary to maintain appropriate distance from X‐ray unit during procedures. However, the endoscopist must stand somewhere close to the X‐ray unit to successfully perform the procedure. It is important to not approach the X‐ray unit unless required and to be careful not to take fluoroscopic images when the nurse approaches the X‐ray unit due to body movement or intravenous drugs.

### Time

The FT and radiation exposure were proportional. The shortest possible FT is recommended for any procedure in FGEPs. Unnecessarily prolonged FT, obtaining many unintended X‐ray images, leads to a substantial increase in both patient and occupational exposure. There are several ways to reduce the FT for FGEPs.

The first is to shorten the FT to the maximum extent possible by doing whatever can be evaluated before the procedure.

The second is for endoscopists to gain knowledge and experience. It has been reported that the FT is shortened when ERCP is performed by an experienced endoscopist. Compared with endoscopists who performed >200 ERCPs in the previous year, endoscopists who performed <100 and 100–200 ERCPs in the previous year had 59% and 11% more FTs, respectively. For every 10‐year experience, the FT was decreased by 20%.^42^

The third is by setting a limit on the PT, which may reduce both patient and occupational exposures by significantly decreasing the FT.^43^

### Shielding

With respect to shielding, shields from X‐ray units and fully protective clothing for medical personnel (protective aprons, thyroid shields, and lead glasses) can be used.

Acrylic shields, equivalent to 0.5‐mm lead, are well‐known radiation shields and have been reported to reduce occupational exposure by a factor of 11.^38^

**Figure 4:**
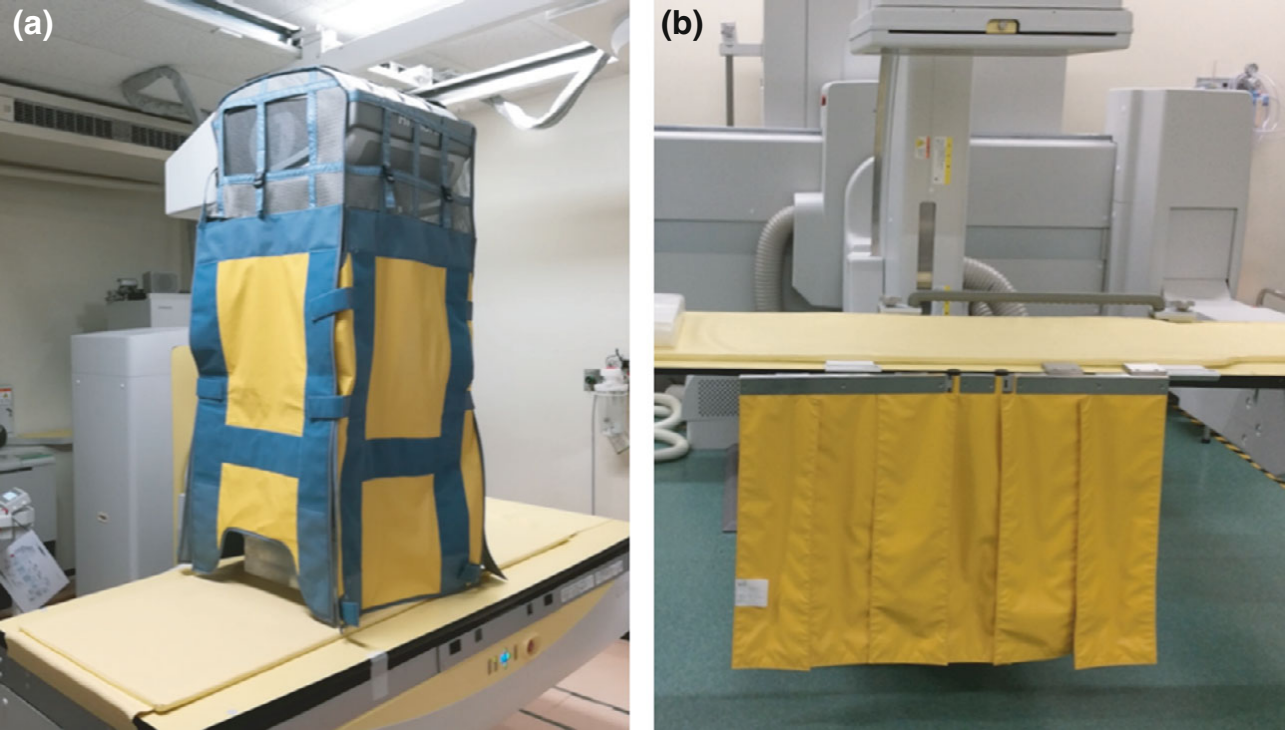
Protective lead shields. (a) For over‐couch tube X‐ray unit. (b) For under‐couch tube X‐ray unit.

Protective lead shields for an under‐couch tube X‐ray unit have been reported, and in recent years, the usefulness of protective lead shields for over‐couch tube types has also been reported (Fig. 4).^35^, ^44^ Protective lead shields for the over‐couch tube type reduced the scattered radiation by up to 89.1% in a phantom study.^45^ Similarly, the radiation dose at the endoscopist's position was reported to be reduced by up to 97%.^44^

However, when the shield is rolled up due to unstable patient sedation or vital signs, the scattered radiation cannot be protected. In such situation, nurses and other personnel will also be close to the X‐ray unit, creating a dangerous situation that increases radiation exposure. In addition, even if shields are used, scattered radiation cannot be completely reduced. Even with the use of a protective lead shield for the over‐couch tube X‐ray, the measured eye lens dose for the endoscopist standing closest to the X‐ray unit was close to 20 mSv/year.^46^ This result is an important reminder that occupational exposure protection requires the proper use of both X‐ray shielding and protective clothing.

Longer working hours have been reported to be associated with higher incidence of radiation‐induced cataract related to ERCP, and protective glasses are strongly recommended.^47^

**Figure 5:**
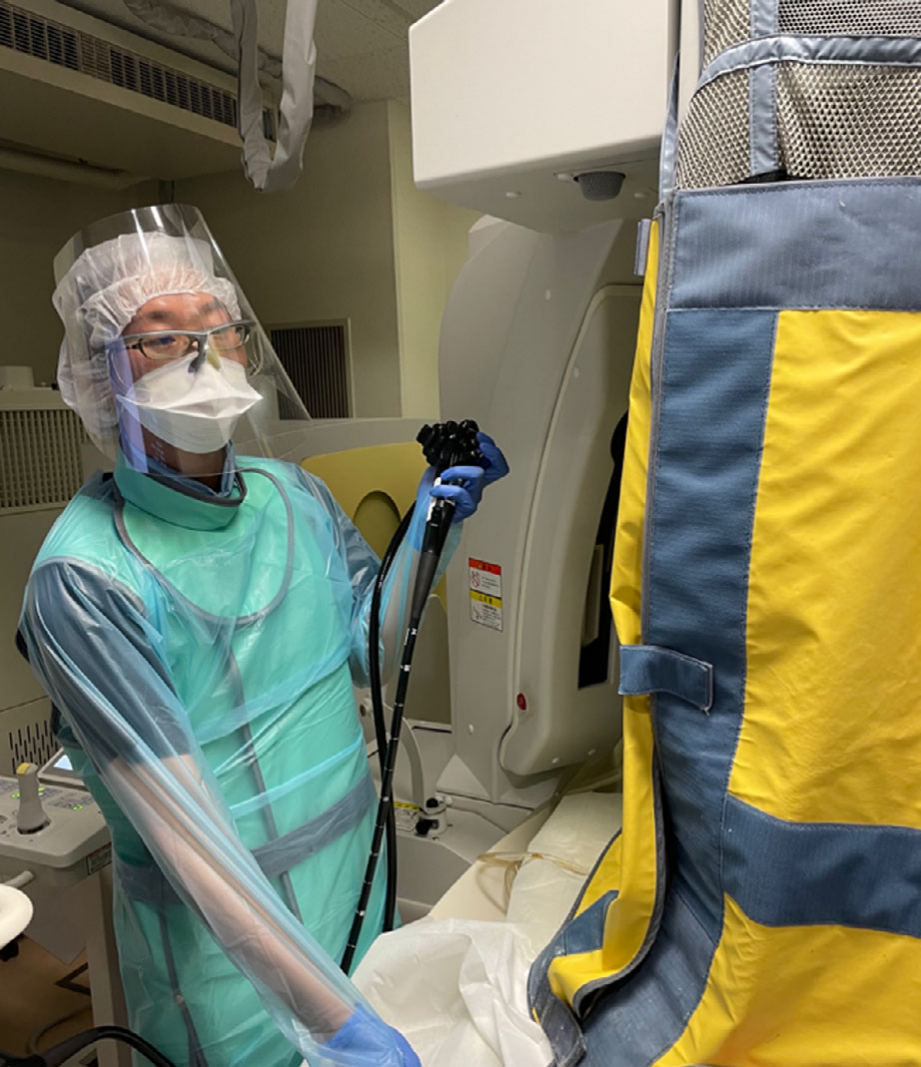
Appropriate radiation protection measures to be taken by endoscopists. This endoscopist performed endoscopic retrograde cholangiopancreatography using an over‐couch tube X‐ray unit with protective lead shields. He wore a radiation protection apron around his neck and body, radiation protection glasses on top of his regular glasses, and a coronavirus disease 2019 protective face shield.

However, many gastroenterologists do not routinely wear protective clothing during FGEP examinations. Surprisingly, the wearing rate of endoscopists was reported to be 27% for a thyroid shield and 21% for lead glasses.^48^ This indicates that gastroenterologists are regrettably still insufficiently aware of radiation protection. In addition, with the advent of the coronavirus disease 2019 (COVID‐19), endoscopists are also required to wear face guards to protect against infection during procedures (Fig. 5).^49^, ^50^, ^51^, ^52^, ^53^, ^54^, ^55^ Rather than choosing between infection protection and exposure protection, endoscopists must take a stance that always protects from both.

## Ingenuity to Reduce Radiation Dose

Recently, the development of equipment has played an important role in reducing radiation dose in FGEPs.

As the latest review described, the current consensus requires imaging device manufacturers to urgently develop imaging technologies that are safer for patients.^56^

The grid‐controlled pulsed fluoroscopy unit has been reported to achieve significantly lower patient doses without the loss of diagnostic accuracy for various abdominal and pelvic fluoroscopic examinations.^57^ The image processing technique of frame rate conversion (FRC) can provide images at a frame rate twice the X‐ray pulse rate by interpolating from two consecutive fluoroscopic images. If the X‐ray pulse rate is 6.25 pulses per second, the FRC provides 12.5 frames per second images. This FRC also has been reported to significantly reduce the radiation dose without extending the FT and maintaining the image quality compared with the conventional method.^58^

A single‐center observational study of radiation dose used in ERCP over an 8‐year period reported that the radiation dose was lower after fluoroscopy device updates.^59^, ^60^

Furthermore, in recent years, a new technology called “spot fluoroscopy” has been developed, in which the fluoroscopic image of the entire abdomen is displayed as a still image on the monitor screen, and only the area where the procedure is being performed, such as the tip of the guidewire, can be observed in real time and moved within the still image.^61^ This technology can reportedly perform procedures with reduced exposure dose compared with conventional fluoroscopy.^62^, ^63^

In addition, there have been many reports in the pediatric urology department to reduce the exposure of pediatric patients, which indicates the need to reduce the exposure of endoscopists.

For example, there have been reports on the following: (i) less exposure of a flat panel detector than an image intensifier,^64^ (ii) comparison of radiation exposure from fixed table fluoroscopy to a C‐arm during ureteroscopy,^65^ (iii) significant reduction of radiation dose with introduction of a checklist,^66^ and (iv) usefulness of pulse fluoroscopy.^67^ These reports have been increasing in recent years and may be helpful for future exposure prevention in the field of gastroenterology.

It is expected that technological advancements aimed at reducing radiation exposure will continue in the future, and gastroenterologists are required to constantly strive to be aware of these improvements.

## Conclusion

Gastroenterologists who perform FGEPs are required to protect not only themselves but also the patients, as well as the nurses and radiologists engaged in examinations, from radiation exposure. Therefore, knowledge about radiation exposure needs to be gained and constantly uploaded based on the latest versions.

## Conflict of Interest

Authors declare no conflict of interest for this article.

## Funding information

None.
